# Supplementary material for: Evaluation of a Novel Fixative Solution for Liquid-Based Cytology in Diagnostic Cytopathology
Source: Diagnostics (Basel). 2023 Dec 5;13(24):3601. doi: 10.3390/diagnostics13243601 (PMC10742394; doi:10.3390/diagnostics13243601)
Supplement: Supplementary file 1 [file diagnostics-13-03601-s001.zip › diagnostics-2705410-supplementary.pdf]

SUPPLEMENTARY MATERIALS

Table S1. Assessment of dichotomized features with categories excellent vs non-excellent (2 vs [0+1]) on gynecological samples. *the reported p-values are the unadjusted one. However, method comparison adjusted for diagnosis was performed and no effect of this variable was found.

	**New (N=139)**	**Std (N=139)**	**P-value ***
**OVERALL DIAGNOSTIC ADEQUACY**			
**0+1**	13 (9.4%)	15 (10.8%)	0.789
**2**	126 (90.6%)	124 (89.2%)	
**OVERALL QUALITY CYTOLOGIC SLIDE**			
**0+1**	19 (13.7%)	28 (20.1%)	0.110
**2**	120 (86.3%)	111 (79.9%)	
**SPOT**			
**0+1**	12 (8.6%)	6 (4.3%)	0.211
**2**	127 (91.4%)	133 (95.7%)	
**CELLULARITY**			
**0+1**	40 (28.8%)	39 (28.1%)	0.997
**2**	99 (71.2%)	100 (71.9%)	
**BACKGROUND**			
**0+1**	16 (11.5%)	61 (43.9%)	<0.001
**2**	123 (88.5%)	78 (56.1%)	
**CELL CLUSTERS**			
**0+1**	68 (48.9%)	31 (22.3%)	<0.001
**2**	71 (51.1%)	108 (77.7%)	
**TOT_score**			
**<12**	110 (79.1%)	117 (84.2%)	0.349
**12**	29 (20.9%)	22 (15.8%)	

Table S2. Assessment of dichotomized features with categories excellent vs non-excellent (2 vs [0+1]) on non-gynecological samples. *the reported p-values are the unadjusted one. However, method comparison adjusted for diagnosis was performed and no effect of this variable was found.

	N (N=183)	C (N=183)	P-value *
**OVERALL DIAGNOSTIC ADEQUACY**			
**0+1**	19 (10.4%)	16 (8.7%)	0.579
**2**	164 (89.6%)	167 (91.3%)	
**OVERALL QUALITY CYTOLOGIC SLIDE**			
**0+1**	30 (16.4%)	54 (29.5%)	0.001
**2**	153 (83.6%)	129 (70.5%)	
**SPOT**			
**0+1**	9 (4.9%)	93 (50.8%)	<0.001
**2**	174 (95.1%)	90 (49.2%)	
**CELLULARITY**			
**0+1**	108 (59.0%)	104 (56.8%)	0.689
**2**	75 (41.0%)	79 (43.2%)	
**CELL CLUSTERS**			
**0+1**	19 (10.4%)	10 (5.5%)	0.080
**2**	164 (89.6%)	173 (94.5%)	
**TOT_score**			
**<10**	124 (67.8%)	131 (71.6%)	0.457
**10**	59 (32.2%)	52 (28.4%)	
